# Traditional food and herbal uses of wild plants in the ancient South-Slavic diaspora of Mundimitar/Montemitro (Southern Italy)

**DOI:** 10.1186/1746-4269-8-21

**Published:** 2012-06-06

**Authors:** Alessandro di Tizio, Łukasz Jakub Łuczaj, Cassandra L Quave, Sulejman Redžić, Andrea Pieroni

**Affiliations:** 1 University of Gastronomic Sciences, Piazza Vittorio Emanuele 9, I-12060, Pollenzo Cuneo, Italy; 2 Department of Ecotoxicology, Faculty of Biotechnology, University of Rzeszów, Werynia 502, 36-100, Kolbuszowa, Poland; 3 Center for the Study of Human Health, Emory University, 550 Asbury Circle, Candler Library 107, Atlanta, GA 30322, USA; 4 Centre of Ecology and Natural Resources, Faculty of Science, University of Sarajevo, 33-35 Zmaja od Bosne St., 71 000, Sarajevo, Bosnia and Herzegovina

**Keywords:** Ethnobotany, Wild food plants, Montemitro, Molise-Slavic, Molise

## Abstract

**Background:**

In Europe, only a limited number of cross-cultural comparative field studies or meta-analyses have been focused on the dynamics through which folk plant knowledge changes over space and time, while a few studies have contributed to the understanding of how plant uses change among newcomers. Nevertheless, ethnic minority groups and/or linguistic “isles” in Southern and Eastern Europe may provide wonderful arenas for understanding the various factors that influence changes in plant uses.

**Methods:**

A field ethnobotanical study was carried out in Mundimitar (Montemitro in Italian), a village of approx. 450 inhabitants, located in the Molise region of South-Eastern Italy. Mundimitar is a South-Slavic community, composed of the descendants of people who migrated to the area during the first half of the 14^th^ century, probably from the lower Neretva valley (Dalmatia and Herzegovina regions). Eighteen key informants (average age: 63.7) were selected using the snowball sampling technique and participated in in-depth interviews regarding their Traditional Knowledge (TK) of the local flora.

**Results:**

Although TK on wild plants is eroded in Montemitro among the youngest generations, fifty-seven taxa (including two cultivated species, which were included due to their unusual uses) were quoted by the study participants. Half of the taxa have correspondence in the Croatian and Herzegovinian folk botanical nomenclature, and the other half with South-Italian folk plant names. A remarkable link to the wild vegetable uses recorded in Dalmatia is evident. A comparison of the collected data with the previous ethnobotanical data of the Molise region and of the entire Italian Peninsula pointed out a few uses that have not been recorded in Italy thus far: the culinary use of boiled black bryony (*Tamus communis*) shoots in sauces and also on pasta; the use of squirting cucumber ( *Ecballium elaterium*) juice for treating malaria in humans; the aerial parts of the elderberry tree ( *Sambucus nigra*) for treating erysipelas in pigs; the aerial parts of pellitory ( *Parietaria judaica*) in decoctions for treating haemorrhoids.

**Conclusions:**

The fact that half of the most salient species documented in our case study – widely available both in Molise and in Dalmatia and Herzegovina – retain a Slavic name could indicate that they may have also been used in Dalmatia and Herzegovina before the migration took place. However, given the occurrence of several South-Italian plant names and uses, also a remarkable acculturation process affected the Slavic community of Montemitro during these last centuries. Future directions of research should try to simultaneously compare current ethnobotanical knowledge of both migrated communities and their counterparts in the areas of origin.

## Introduction

One of the most intriguing scientific questions in ethnobiology concerns the ways through which folk plant knowledge changes over space and time. In Europe, only a limited number of cross-cultural comparative field studies or meta-analyses of historical ethnobotanical literature focused on such dynamics so far [1-8], while an increasing number of studies have contributed to the understanding of how plant uses change among “newcomers” [9-17].

Ancient linguistic diasporas have instead been the focus of several field ethnobotanical surveys in Italy during the last decades. Studies on Traditional Knowledge (TK) of plant uses have thus far involved a number of ethnic minority groups within the Italian geographical region: in Northern Italy, Occitans (Provençal) [18-23], Franco-Provençal [20,24-26] and German Walser [27-29] groups in Piedmont, Ladins [30-33], Mócheno [34] and Cimbrian [35,36] Bavarians in Veneto and Trentino; Istro-Romanians in the Croatian Istria [37]; in Southern Italy: Albanian Arbëreshë in Lucania [38-40] and Greeks in Calabria [41,42]; in Sardinia, Tabarkins (Ligurians) [43].

The present study focused on the food and herbal ethnobotany of an ancient South-Slavic diaspora living in the village of Mundimitar/Montemitro, Molise Region, South-Eastern Italy.

The aims of this study were:

· to record folk food and herbal uses of wild plants and mushrooms in Mundimitar;

· to compare the collected ethnolinguistic data with those of Molise, surrounding Italian regions and of Croatia and Herzegovina;

· to compare the recorded ethnobotanical uses with all Italian ethnobotanical literature;

· to assess the resilience and cultural adaptations of the Slavic diaspora in perceptions (naming) and uses of wild plants.

## Methodology

### Study site

**Figure 1:**
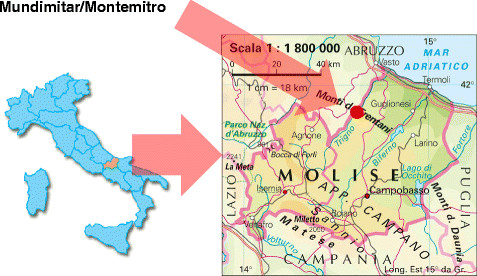
Location of Mundimitar/Montemitro.

Mundimitar (in Italian Muntemitro) is a small village located at 508 m.a.s.l. in the Province of Campobasso, Molise Region, Southern Italy (Figure 1).

Like Acquaviva Collecroce and San Felice del Molise, Montemitro is the home to a Slavic community that migrated in the area, probably from the lower Neretva valley (Dalmatia and Herzegovina regions) during the first half of the 14^th^ century [44].

The village had a population of ca. 1,000 inhabitants until the 1970’s when many locals migrated to Northern Italy or abroad for employment. Nowadays, the village is composed of ca. 450 inhabitants who speak a Western Štokavian dialect (*na-našo* in the local language, meaning “in our language”), known by linguists as Molise Slavic or Molise Croatian.

### Field study

The field ethnobiological study was carried out in Mundimitar during several visits in 2009 and 2010. Eighteen key informants (average age: 63.7) were selected using snowball sampling techniques and participated in in-depth interviews regarding their TK of the local flora. The focus of the interviews was on folk food and medicinal uses of wild food plants and mushrooms. Prior informed consent (PIC) was obtained verbally before commencing each interview and the guidelines of the AISEA (Italian Association for Ethno-Anthropological Sciences) [45] were adhered to.

Free-listing and semi-structured interview techniques were used. When available, the wild plant species cited during interviews were collected, verified by our interviewees, identified according to Pignatti’s *Flora d’Italia*[46], named according to Tutin et al.’s *Flora Europaea*[47] and later deposited at the Herbarium of the University of Gastronomic Sciences in Pollenzo. Plant family names follow the recent classification (III) of the Angiosperm Phylogeny Group. The local folk plant names cited during interviews were recorded and transcribed in Serbo-Croatian (after the Yugoslav dissolved, also named BCSM – Bosnian/Bosniak-Croatian-Serbian-Montenegrin).

### Data analysis

The data collected during the field study were sorted in Microsoft® Excel.

Two in-depth comparisons were conducted:

· the former, concerning folk plant names, with the standard work on Croatian and Italian folk phytonimy [48,49], a comprehensive review of the food ethnobotany of Abruzzo (the Italian region bordering Molise) [50], and unpublished ethnobotanical data of SR from Herzegovina as well as the unpublished list of wild food plants sold in the main eleven Dalmatian vegetable markets in March 2012 (ŁŁ);

· the latter, concerning the folk plant uses, with the most comprehensive review of the Italian ethnobotany (published in 2006) [51] and a few additional recent ethnobotanical field studies conducted in the Molise region [52-55]. Data from the studied village concerning wild green vegetables were compared with Croatian literature concerning plants use in Dalmatia [56-59] and with personal (ŁŁ ) observations on wild vegetables sold in Dalmatian markets in 2012.

## Results and discussion

Table 1 shows the local food and medicinal uses of wild vascular plants and mushrooms recorded in Montemitro. Fifty-seven species were identified by study participants. The table includes also the unusual food uses of two cultivated species (garlic and lupine). The limited number of identified fungi is attributed to the fact that most of the cited folk names did not occur during the visit in the field and could not be clearly identified.

**Table 1:** Traditional food and medicinal uses of wild plants and mushrooms in Mundimitar/Montemitro

**Botanical taxon and family**	**Local name(s) in Mundimitar**	**English name**	**Part(s) used**	**Folk use(s) in Mundimitar**	**Frequency of citation**
*Allium sativum* L. (Amaryllidaceae) (CULTIVATED)	Luk	Garlic	Flowering shoots	Boiled, then preserved in olive oil or vinegar; in tomato sauces	+++
*Amaranthus retroflexus* L. (Amaranthaceae)	Pjedruš	Amaranth	Leaves	Raw in salads, or boiled	+++
*Apium nodiflorum* (L.) Lag. (Apiaceae)	Kanijola	Fool's water-cress	Aerial parts	Raw in salads or between two slices of bread	+++
*Armillaria mellea* (Vahl) P. Kumm and related species (Marasmiaceae)	Rekkie mušil	Honey fungus	Fruiting body	Blanched, then fried	+
*Asparagus acutifolius* L. (Asparagaceae)	Sparuga	Wild asparagus	Shoots	Boiled, then fried in omelets	+++
*Beta vulgaris* subsp. *maritima (*L.) Arcang. (Amaranthaceae)	Blitva	Wild beet	Leaves	Boiled, then fried	+++
*Borago officinalis* L. (Boraginaceae)	Bureina	Borage	Young leale	Boiled.	+++
				Coated with bread crumbs, then deep fried	
*Bunias erucago* L. (Brassicaceae) (?)	Rapanača	Crested warty cabbage	Whorls	Boiled and fried	+
*Calendula arvensis* L. (Asteraceae)	Kalendula	Marigold	Flowers	In salads	+
*Cantharellus cibarius* Fr. (Cantharellaceae)	Galuč	Chanterelle	Fruiting body	Blanched, then fried	+
*Centaurium erythraea* Rafn. (Gentianaceae)	Džencjanela	Centaury	Aerial parts	Decoction as a panacea	+
*Cichorium intybus* L. (Asteraceae)	Čikoria	Wild cichory	Whorls	Boiled, then fried in olive oil with garlic	++
*Clavaria* sp. (Clavariaceae)	Picele	Coral fungus	Fruiting body	Boiled, then fried	+
*Clematis vitalba* L. (Ranunculaceae)	Škrabut	Traveller’s joy	Shoots	Boiled, then fried or in sauces; digestive aid	+++
				Stems are directly applied on the tooth to treat toothache	
*Cornus mas* L. (Cornaceae)	Kurnja	Cornel cherry tree	Fruits (*Kurnjal*)	Consumed raw, or dried/smoked; liqueurs	+++
*Crataegus. monogyna* Jacq. and *C. oxyacantha* L. (Rosaceae)	Glog	Hawthorn	Fruits (*Glogbili*)	Consumed raw as snack.	+++
				The thorny stems were used to insert into figs for drying.	
*Cydonia oblonga* Mill. (Rosaceae)	Kutunja	Quince	Fruits	Boiled with wine, for treating sore throats.	+++
				Jam.	
*Cynara cardunculus* L. (Asteraceae)	Ošnak	Wild artichocke or wild cardoon	Stems	Boiled, then fried with eggs	+++
*Cynodon dactylon* (L.) Pers. (Poaceae)	Gramača	Bermuda grass	Whole plant	Decoction as a diuretic	++
*Diplotaxis erucoides* (L.) DC. (Brassicaceae)	Marijun	White wall-rocket	Leaves	Raw in salads, more often fried in the pan	+++
*Ecballium elaterium* (L.) A. Rich. (Cucurbitaceae)	Tikvica divlja	Squirting cucumber	Fruit juice	Instilled in the nose for treating malaria or spread on women breast for weaning babies	++
*Eruca sativa* Miller (Brassicaceae)	Rucola	Rocket	Leaves	Raw in salads	+++
*Eryngium campestre* L. (Apiaceae) (?)	Sikavac	Field eryngo	Leaves	Decoction for treating eye inflammations	+
*Ficus carica* L. (Moraceae)	Smokva	Fig tree	Pseudofruits	Eaten fresh or dried	+++
*Foeniculum vulgare* Mill. subsp. *piperitum* (Ucria) Cout. (Apiaceae)	Finoč	Wild fennel	Fruits	Seasoning for home-made sausages; decoctions as diuretic or for treating gastric reflux	+++
*Glycyrrhiza glabra* L. (Fabaceae)	Gurgulica	Licorice	Root	Consumed raw as snack.	+++
				The aerial parts used as insect repellent.	
*Humulus lupulus* L. (Cannabaceae)	Lupare	Wild hop	Shoots	Boiled, then fried in omelet	++
*Hydnum repandum* L.: Fr. (Hydnaceae)	Lengaove	Wood hedgehog	Fruiting body	Blanched, then fried	++
*Lupinus albus* L. spp. (Fabaceae) (CULTIVATED)	Lupino	Lupin	Flower shoots Aerial parts	Boiled, then fried.	+
				The decoction of the whole aerial parts is used in external washes for treating pig erysipelas	
*Malva sylvestris* L. (Malvaceae)	Slis	Mallow	Leaves and flowers	Decoction for treating digestive troubles, bronchitis, or as a laxative for children	+++
*Matricaria chamomilla* L. (Asteraceae)	Kamomilla	Chamomile	Flowering tops or stems	Decoction, as a mild tranquillizer	++
*Mercurialis annua* L. (Euphorbiaceae)	Merkulela	Mercurya	Leaves	Boiled in soups (mixed with other herbs), or in purgative decoctions	++
*Olea europaea* L. var. *sylvestris* Brot. (Oleaceae)	Maslina	Wild olive tree	Branches	Used for drying figs	++
*Origanum vulgare* L. (Lamiaceae)	Pljei	Wild oregano	Flowering tops	Seasoning	+++
*Papaver rhoeas* L. (Papaveraceae)	Mak	Corn poppy	Young aerial parts	Raw in salads, or cooked	+++
*Parietaria judaica* L. (Urticaceae)	Kolana	Pellitory	Aerial parts	Decoction in external use for treating hemorrhoids (affected parts exposed to vapors).	++
				Necklaces for children	
*Picris echioides* L. and *P. hieracioides* L. (Asteraceae)	Tustača	Oxtongue	Whorls and shoots	Shoots eaten raw as snack.	++
				Whorls boiled and fried.	
*Portulaca oleracea* L. (Portulacaceae)	Prkatj	Purslane	Aerial parts	Raw in salads	++
*Prunus spinosa* L. (Rosaceae)	Ndrnjela	Sloe	Fruits	Gathered an consumed after the frost; liqueurs	++
*Punica granatum* L. (Punicaceae)	Šipak	Pomegranate	Fruits	Consumed raw in winter	++
*Pyrus pyraster* Burgsd. (Rosaceae)	Trnovača	Wild pear tree	Fruits	Gathered and consumed after the frost	++
*Quercus virgiliana* (Ten.) Ten. (Fagaceae) (?)	Sladul	Oak	Kernel	Consumed raw	+
*Rosa canina* L. (Rosaceae)	Skorčavata	Dog rose	Pseudofruits	Decoction for treating sore throat (sometimes together wild dried figs, apple slices, and barley)	+++
*Ruscus aculeatus* L. (Asparagaceae)	Leprencia	Butcher’s Broom	Shoots	Boiled, then fried.	++
				Dried branches were used to clean the fireplace	
*Ruta graveolens* L. (Rutaceae)	Ruta	Rue	Aerial parts	Aromatizing grappa.	+++
				Kept under the pillow for treating worms in children.	
				A few leaves eaten raw by pregnant women to prevent miscarriage (in the past)	
*Salvia verbenaca* L. (Lamiaceae)	Prsenica	Meadow sage	Leaves	Applied externally with pork fat as a suppurative or for treating insect stings	+
*Sambucus nigra* L. (Caprifoliaceae)	Baz	Elderbery tree	Aerial parts and fruits	Decoction, then in external washes for treating erysipelas in pigs.	+++
				Fruits juice used as ink in the past.	
*Sinapis alba* L and *S. arvensis* L. (Brassicaceae)	Sinapa	Wild mustard	Young aerial parts	Raw in salads, more often cooked in the pan	++
*Sonchus arvensis* L. and *S. oleraceus* L. (Asteraceae)	Kostriš/ Kašgn	Sow thistle	Young aerial parts	Boiled, then fried in the pan or cooked in tomato sauce	+++
*Sorbus domestica* L. (Rosaceae)	Oskoruša	Service tree	Fruits	Consumed after natural fermentation	++
*Stellaria media* (L.) Vill. (Caryophyllaceae)	Mišakina	Chickweed	Aerial parts	Fodder for hens	++
*Tamus communis* L. (Dioscoreaceae)	Gljuštre	Black bryony	Shoots	Boiled, then fried in the pan with eggs or tomato sauce (sometimes served on noodles)	+++
*Teucrium chamaedrys* L. (Lamiaceae)	Kametr	Wall germander	Aerial parts	Decoction for treating malaria (in the past) and hypertension	++
*Umbilicus rupestris* (Salisb.) Dandy (Crassulaceae)	Kopič	Navelwort	Leaves	Crushed and mixed with pork fat and soot for treating furuncles	++
*Urtica dioica* L (Urticaceae)	Kopriva	Nettle	Leaves and shoots	Boiled, then mixed with ricotta cheese, in filled pasta.	+++
				Decoction in external washes for strengthening the hair	
*Ziziphus jujuba* Miller (Rhamnaceae)	Džurdžula	Jujube	Fruits	Eaten after natural fermentation	+

(?) Identification was only postulated on the basis of linguistic data and plant description; +++: quoted by 7 informants or more; ++: quoted by 2 to 6 informants; +: quoted by 1 or 2 informants only.

**Figure 2:**
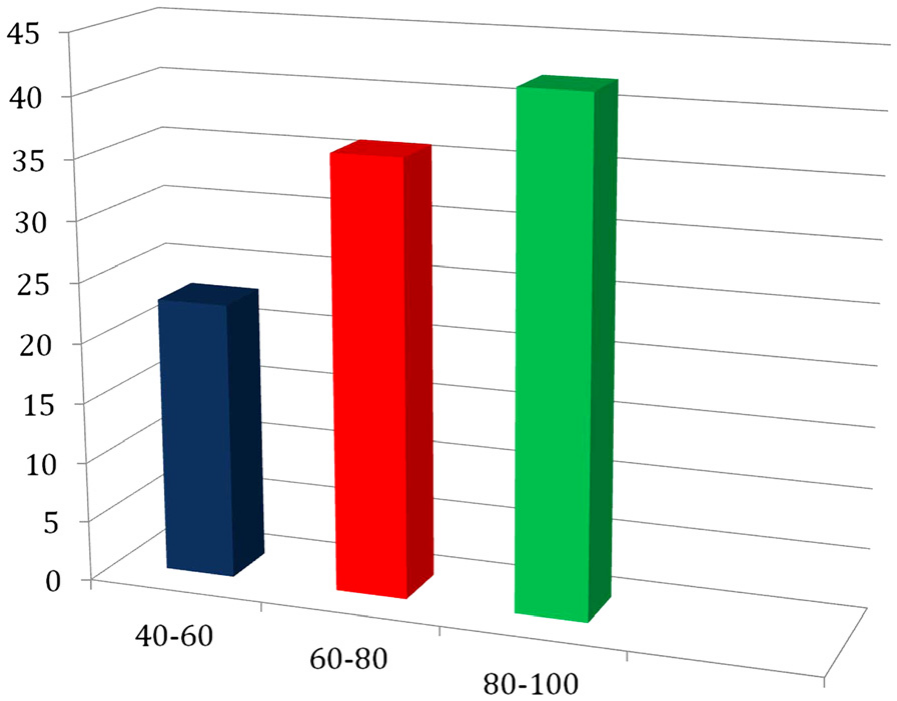
Average number of species quoted by informants by age.

Figure 2 shows that TK on wild plants is eroded in Montemitro among the youngest generations, thus confirming trends that are the similar throughout Southern Europe and in a large part of the world.

Table 2 shows the ethnolinguistic comparative analysis of the most quoted species during the free-listing exercise (quoted by more than 40% of the informants).

**Table 2:** Ethnolinguistic analysis of the most quoted wild food plants in Mundimitar/Montemitro (linguistic correspondences are underlined)

**Botanical taxon**	**Local name (s) in Mundimitar**	**Folk name (s) in Croatia**[48]**and Herzegovina (unpublished data)**	**Folk name (s) in Molise and surrounding South Italian regions**[49,50,52,59]
*Amaranthus retroflexus*	Pjedruš	Lodoba, Štir	Cime de halle, Pricacchione, Pederosse
*Apium nodiflorum*	Kanijola	Celer	Candele, Cannizzole, Cannole, Lacce selvagge, Sellarina
*Asparagus acutifolius*	Sparuga	Sparožin, Šparoga	Sparacane, Sparaci, Sparge, Sperne, Spinele
*Beta vulgaris*	Blitva	Bitva divja, Blitva divja, Cikla	Biete, Biote
*Borago officinalis*	Bureina	Borač, Boražina	Burracce, Burraina, Verraina
*Clematis vitalba*	Škrabut	Pavina, Pavit, Škrobut	Vitavale, Vitelle, Vitacchie
*Cornus mas*	Kurnja	Drijen, Drin	Corniale, Crugnare, Vrignale
*Crataegus monogyna/C. oxyacantha*	Glog	Glog	Arciprande, Bianghespine, Ciciarille, Spine bianghe
*Cynara cardunculus*	Ošnak	Artičok, Gardun	Cardone, Carducce, Scalelle
*Foeniculum vulgare*	Finoč	Komorač, Mirodjija, Morač	Fenucchie
*Mercurialis annua*	Mrkulela	Resulja, Šcerenica	Mercorella, Murculella
*Origanum vulgare*	Pljei	Metvica, Mravinac, Vranilovka, Vranilova trava	Arigano, Pnliejo, Regana
*Papaver rhoeas*	Mak	Bologlav, Mak divlji	Papaina,Papambele, Pupille
*Portulaca oleracea*	Prkatj	Štucliak, Tušani, Tušt	Perchiacche, Porcacchie, Precacchie
*Prunus spinosa*	Ndrnjela	Brombuli, Crni trn, Trnjina	Ndrignazze, Prugnele, Spine perugne, Struzzacane
*Rosa canina*	Skorčavata	Srbiguz, Šipak, Šipurak,	Cacavescie, Raspacule, Scarciacule, Stracciacule
*Sinapis* spp.	Sinapa	Gorušica, Muštarde	Lassane, Sinape
*Sonchus* spp.	Kostriš/ Kašgn	Kostriš, Ostak, Mličika, Slatčica	Cascigne, Crespigne, Respigne
*Sorbus domestica*	Oskoruša	Oskoruša	Ciorve, Scioreve
*Tamus communis*	Gljuštre	Bljušt	Afine, Curone, Defano
*Urtica dioica*	Kopriva	Bažgava, Kopriva, Žara	Ardiche, Arteche, Strica

Half of the taxa have correspondence in the Croatian and Herzegovinian folk botanical nomenclature, and the other half with South-Italian folk plant names.

The most quoted species still retain a Slavic name and may have also been used in Dalmatia and Herzegovina before the migration took place. A similar link between linguistic cognates and cultural salience has been shown in a recent food ethnobotanical study conducted among a Greek minority in Calabria [41]. However, this analysis may only express a reasonable probability of the original permanence of plant uses into a new environmental and cultural space, but it cannot be excluded that migrant groups may have acquired new practices of use of previously known plants from the autochthonous population, thus resulting in naming plants with the original language and using them in a very different way from the original one.

On the other hand, the fact that in our case study half of the most salient species – widely available both in Molise and in Dalmatia and Herzegovina-- have South-Italian folk names demonstrates a strong acculturation process that has affected the Slavic community of Montemitro during these last centuries.

We have also compared our findings with all of the previous ethnobotanical data of the Molise Region and of the entire Italian Peninsula. A few uses seem to have been recorded for the first time:

· the culinary use of boiled black bryony (*Tamus communis*) shoots in sauces and also with pasta;

· the use of squirting cucumber (*Ecballium elaterium*) juice for treating malaria in humans (in the past);

· the use of aerial parts of the elderberry tree (*Sambucus nigra*) for treating erysipelas in pigs;

· the use of decoctions of pellitory (*Parietaria judaica*) for treating haemorrhoids.

Specific ethnobotanical surveys conducted in Dalmatia and Herzegovina are missing in the literature, thus making it very difficult to draft a comprehensive comparison on plant uses. However, a food use of black bryony shoots, which is very common in Mundimitar, seems to be nowadays also common in the Istrian cuisine in Croatia [60] as well in Dalmatia, where it is commonly sold in markets (ŁŁ personal observation, 2012), while in Molise and Abruzzo its food use is considered obsolete.

The wild vegetable mix called *mišanca* (Zadar-Split) */pazija* (Dubrovnik) *,* sold in every market of the Dalmatian coast (surveyed in March 2012, Łuczaj unpubl.) contains many of the plants used in the study area. For example *Sonchus* spp., *Foeniculum vulgare, Papaver rhoeas, Picris echioides,* and to a much lesser extent *Eryngium* sp. are used as food in Dalmatia nowadays (Table 3). However the existence of this concept of vegetable mix was not recorded in the study area. Strikingly, the data from the study area contain relatively few Asteraceae species, nowadays widely used in Dalmatia under the name *radič* or *žutenica* (e.g. *Taraxacum* spp. *, Crepis* spp.) and a few other related genera). It must be kept in mind that Dalmatia was under a strong Greek, Roman and Venetian influence, and that the practice of using a variety of wild vegetables in Dalmatia may have a non-Slavic origin. Thus it may be that some of the uses brought by the Slavic emigrants to Italy are actually re-imports of Venetian or Latin customs.

**Table 3:** Comparison of the use of wild green vegetables in Mundimitar with the studies from Dalmatia and Hercegovina (the areas where the diaspora of Mundimitar originated)

	**Use in the W Balkans**	**Use in Mundimitar**
*Amaranthus retroflexus* L. (Amaranthaceae)	G	x
*Apium nodiflorum* (L.) Lag. (Apiaceae)		x
*Asparagus spp.* (mainly *Asparagus acutifolius* L. ) (Asparagaceae)	B, G, C, M	x
*Beta vulgaris* L. (Amaranthaceae)	B, G	x
*Borago officinalis* L. (Boraginaceae)	G, S	x
*Bunias erucago* L. (Brassicaceae)	G	x
*Cichorium intybus* L. (Asteraceae)	B, G, S	x
*Clematis vitalba* L. (Ranunculaceae)	G	x
*Cynara cardunculus* L. (Asteraceae)		x
*Diplotaxis erucoides* (L.) DC. (Brassicaceae)		x
*Eruca sativa* Miller (Brassicaceae)	B, G	x
*Humulus lupulus* L. (Cannabaceae)		x
*Mercurialis annua* L. (Euphorbiaceae)		x
*Papaver rhoeas* L. (Papaveraceae)	G, C, S, L	x
*Picris echioides* L. (Asteraceae)	L	x
*P. hieracioides L. (Asteraceae)*		x
*Portulaca oleracea* L. (Portulacaceae)	G	x
*Ruscus* spp. (Asparagaceae)	B, G, C	x
*Sinapis alba* L and *S. arvensis* L. (Brassicaceae)		x
*Sonchus spp.* (Asteraceae)	B, G, C, S, L	x
*Tamus communis* L. (Dioscoreaceae)	B, G, S, M	x
*Urtica dioica* L (Urticaceae)	S	x
*Foeniculum vulgare* Mill. (Apiaceae)	B, G, C, S, L	only fruits as seasoning
*Allium ampeloprasum* L. (Liliaceae)	B, G, S, L	
*Anchusa arvensis* (L.) M. Bieb. (Boraginaceae)	C	
*Anchusa* sp. (Boraginaceae)	C	
*Arum italicum* Mill. (Araceae)	B	
*Brassica oleracea* L. (Brassicaceae)	G	
*Capsella bursa-pastoris* L. (Brassicaceae)	G	
*Chenopodium urbicum* L. (Chenopodiaceae)	B	
*Cirsium arvense* L. (Asteraceae)	B, G	
*Crepis* sp. (Asteraceae)	C, L	
*Crepis sancta* (L.) Babc. (Asteraceae)	C	
*Crithmum maritimum* L. (Apiaceae)	B, G	
*Daucus carota* L. (Apiaceae)	B, G, S, L	
*Diplotaxis tenuifolia* (L.) DC. (Brassicaceae)	B, G	
*Erodium cicutarium* (L.) L'Hér. ex Aiton (Geraniaceae)	C	
*Eryngium maritimum* L. and *E. campestre* L. (Asteraceae)	B, G	
*Geranium molle* L. (Geraniaceae)	C	
*Hirschfeldia incana* (L.) Lagr.-Foss. (Brassicaceae)	G	
*Hypochoeris radicata* L. (Asteraceae)	G	
*Lactuca perennis* L. (Asteraceae)	B	
*Lactuca serriola* L. (Asteraceae)	S	
*Leontodon tuberosus* L. (Asteraceae)	B	
*Mentha aquatica* L. (Lamiaceae)	B	
*Ornithogalum umbellatum* L. (Liliaceae)	G	
*Reichardia picroides* (L.) Roth. (Asteraceae)	G, S	
*Ranunculus muricatus* L. (Ranunculaceae)	C	
*Rhagadiolus stellatus* (L.) Gaertn. (Asteraceae)	C	
*Rumex* spp. (Polygonaceae)	G, C	
*Salicornia herbacea* L. (Amaranthaceae)	G	
*Silene latifolia* Poir. (Caryophyllaceae)	L	
*Salvia verbenaca* L. (Lamiaceae)	C	
*Silene vulgaris* (Mch.) Garcke and related species (Caryophyllaceae)	B, G	
*Smilax aspera* L. (Smilacaceae)	G	
*Taraxacum megalorrhizon* (Forssk.) Hand.-Mazz. (Asteraceae)	B	
*Taraxacum officinale* Weber (Asteraceae)	B, G, L	
*Tordylium apulum* L. (Apiaceae)	C	
*Tragopogon pratensis* L. (Asteraceae)	B, G, S	
*Urospermum picroides* (L.) Desf. (Asteraceae)	G, C, L	
*Urtica pilulifera* L. (Urticaceae)	B, G	

B – Bakić and Popović (in this study it is unclear if the data is about eating green parts or underground organs) [56], G – Grlić [57], C – Ćurčić [58], S- Sardelić [59], L – most commonly sold wild greens in Dalmatian markets in 2012 in the form of a vegetable mix (Łuczaj, unpublished); M – sold commonly in Dalmatian markets in 2012 as separate bunches (Łuczaj, unpublished).

It is worth pointing out that nowadays in Dalmatia wild vegetables are mainly boiled, strained and seasoned with olive oil whereas the described uses in the study area often refer to frying, which may be reflective of a more recent acquisition of Italian cooking practices.

The food use of mercury (*Mercurialis annua*) leaves in soups has instead been recorded only one other time in Italy, in two studies conducted in North-Western Tuscany in the Lucca area [61,62].

## Conclusions

This study demonstrates that even within ancient diasporas, as in the Slavic community of Mundimitar, which still exists in Italy after more than five centuries since it was founded, it is possible to find traces of resilience of original TK regarding plants.

A few uses of most quoted plants, which are still named in the original language, may have originated in the migrants’ areas of origin (Dalmatia and Herzegovina).

However, TK is the result of dynamic processes and the case study that we have analysed here also demonstrates a high degree of adaptation, which is shown in both the folk botanical nomenclature (half of the most quoted botanical taxa are named in South-Italian) and in the actual plant folk uses too (very few uses do not correspond with the Italian ethnobotany).

These considerations show that, in contrast with analogous studies conducted on the ethnobotany of recent migrants/newcomer’ groups, TK about plants within ancient diasporas is a very complex, and not well understood, phenomenon.

## Competing interests

The authors declare that they have no competing interests.

## Authors’ contributions

AdT and AP conceived the study and conducted the field study; AP, ŁŁ, SR, and CLQ performed the data analysis and drafted the discussion. All authors read and approved the final manuscript.
